# Control of Intrachain Morphology in the Formation of Polyfluorene Aggregates on the Single‐Molecule Level

**DOI:** 10.1002/cphc.202000118

**Published:** 2020-04-07

**Authors:** Philipp Wilhelm, Dominik Blank, John M. Lupton, Jan Vogelsang

**Affiliations:** ^1^ Institut für Experimentelle und Angewandte Physik Universität Regensburg Universitätsstraße 31 93053 Regensburg Germany

**Keywords:** fluorescence, organic electronics, phase transitions, polyfluorene, single-molecule spectroscopy

## Abstract

Controlling the morphology of π‐conjugated polymers for organic optoelectronic devices has long been a goal in the field of materials science. Since the morphology of a polymer chain is closely intertwined with its photophysical properties, it is desirable to be able to change the arrangement of the polymers at will. We investigate the π‐conjugated polymer poly(9,9‐dioctylfluorene) (PFO), which can exist in three distinctly different structural phases: the α‐, β‐, and γ‐phase. Every phase has a different chain structure and a unique photoluminescence (PL) spectrum. Due to its unique properties and the pronounced spectral structure‐property relations, PFO can be used as a model system to study the morphology of π‐conjugated polymers. To avoid ensemble averaging, we examine the PL spectrum of single PFO chains embedded in a non‐fluorescent matrix. With single‐molecule spectroscopy the structural phase of every single chain can be determined, and changes can be monitored very easily. To manipulate the morphology, solvent vapor annealing (SVA) was applied, which leads to a diffusion of the polymer chains in the matrix. The β‐ and γ‐phases appear during the self‐assembly of single α‐phase PFO chains into mesoscopic aggregates. The extent of β‐ and γ‐phase formation is directed by the solvent‐swelling protocol used for aggregation. Aggregation unequivocally promotes formation of the more planar β‐ and γ‐phases. Once these lower‐energy more ordered structural phases are formed, SVA cannot return the polymer chain to the less ordered phase by aggregate swelling.

## Graphical abstract

**The π‐conjugated polymer** poly(9,9‐dioctylfluorene) (PFO) exists in three different phases: the α‐, β‐, and γ‐phase, which are associated with different chain morphologies. The β‐ and γ‐phases appear during the self‐assembly of single α‐phase PFO chains into mesoscopic aggregates. The extent of β‐ and γ‐phase formation is directed by the solvent‐swelling protocol used for aggregation.

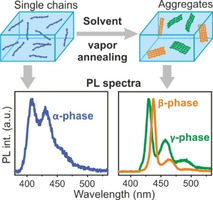

In π‐conjugated polymers the connection between the electronic properties and the morphology of the chain is prominent.^1^, ^2^, ^3^, ^4^, ^5^ A prime example is PFO, for which its morphology can even be separated into distinct phases (the α‐, β‐, and γ‐phase), where each phase has its own very distinct properties.^6^ These phases are purely intramolecular in nature. The β‐phase, for example, has been clearly identified by x‐ray scattering of drawn fibers.^7^ The polymer chain properties have to fit to the desired needs in an optoelectronic device, which are defined by the application. For instance, due to its enhanced charge‐carrier mobility, the β‐phase is most suitable for electronic devices such as transistors.^8^ Unfortunately, simply spin‐coating a PFO film typically results in the formation of the α‐phase, and further treatment must be applied to induce a phase transition to the β‐phase.^9^, ^10^, ^11^ On the one hand, the presence of the β‐phase can be identified easily, because a specific emission band appears in the PL emission spectrum caused by the phase transition. On the other hand, it is impossible to distinguish between different polymer morphologies within the film and therefore a direct connection cannot be drawn between spectroscopic or electronic properties and a certain structural phase. The main reason for this is disorder ensemble averaging, and the fact that energy transfer can occur between different sites within the film, e. g. from the higher‐energy disordered α‐phase to the lower‐energy ordered β‐phase. Disorder and energy transfer therefore need to be suppressed. One way to obtain such a direct connection is by avoiding ensemble averaging and measuring discrete mesoscopic regions of the PFO film. Such an approach can be experimentally achieved by bottom‐up assembly of mesoscopic aggregates, built from one molecule at a time, and investigating these particles with single molecule spectroscopic (SMS) techniques.^12^, ^13^, ^14^

Here, we report manipulation of the PFO phase by applying solvent vapor annealing (SVA) assisted aggregation. During SVA the single PFO polymer chains diffuse inside a swollen non‐fluorescent host matrix and start to aggregate. The aggregation induces an intrachain phase transition, which leads to a change in the emission spectra of the aggregates compared to the single isolated chains. By applying different solvent mixtures, the type of the phase transition can be influenced. Figure 1a provides an overview of the three different phases which PFO can assume.^6^ The phases of PFO differ in their intermonomer torsion angle Φ.^15^ The most common form is the amorphous α‐phase (or glassy‐phase). In this phase, the PFO chains adopt a wormlike geometry with an intermonomer torsion angle randomly distributed around 135°.^15^ In the β‐phase, the torsion angle, Φ, is in the range of 160° to 180°.^16^ This planarization of the polymer backbone leads to a more rigid, linear chain, which gives rise to a red‐shift of the PL emission spectrum by approximately 30 nm compared to the α‐phase.^17^, ^18^ In most cases, only the distinction between these two phases is made. However, an intermediate third stable phase can also occur, the so called γ‐phase. With a torsional angle Φ between 140° and 160°, the γ‐phase features an emission spectrum which is located between the α‐ and the β‐phase.^6^, ^15^ Because of this, the γ‐phase has remained quite elusive and there are only a few reports of its identification in the literature. The phase transitions to either the β‐ or γ‐phase lead to a more linear chain conformation in comparison to the α‐phase. It is crucial to stress here that even though the chains interact physically with each other in the aggregate, unlike molecular crystals or other conjugated polymers such as poly(*para*‐phenylene‐ethynylene‐butadiynylene) (PPEB),^19^ they do not form electronic intermolecular aggregate states such as H‐aggregates.

**Figure 1:**
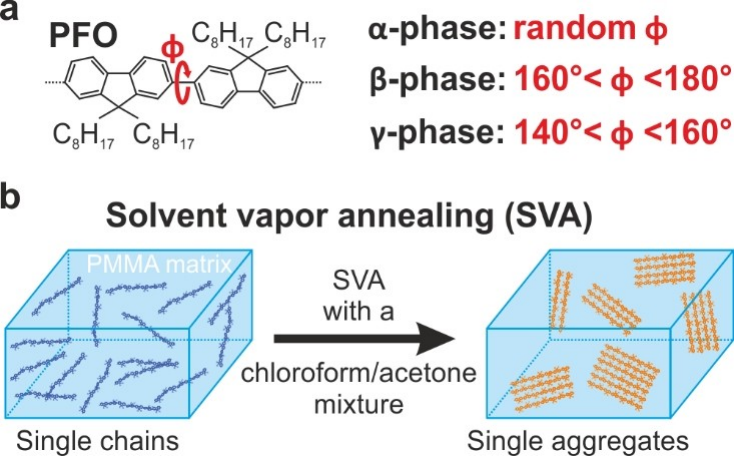
(a) Sketch of the chemical structure of poly(9,9‐dioctylfluorenyl‐2,7‐diyl) (PFO). Depending on the fluorene‐fluorene torsional angle, Φ, PFO can exist in three different conformations (α‐, β‐, and γ‐phase). (b) Inducing phase transitions by aggregation of single polymer chains embedded in a PMMA matrix. Aggregation is controlled by solvent vapor annealing (SVA) with different chloroform/acetone vapor ratios. Note that the emissive species is known to remain intramolecular in nature, i. e. no signatures of H‐aggregation exist.

We have previously illustrated how SVA can be used to aggregate single isolated polymer chains into mesoscopic aggregates with controllable size.^12^ For PPEB, it was shown that the aggregation leads to a more ordered, linear morphology of the polymer chains inside the aggregate.^19^ Building on this result, we aim to use SVA to trigger a phase transition by aggregating single PFO polymer chains. The PFO polymer (ADS129BE; 40–150 kDa) was purchased from American Dye Source, Inc. Figure 1b provides a scheme of the sample preparation. We embedded the polymers in a ∼250 nm thick poly(methyl‐methacrylate) (PMMA) matrix. The PFO concentration in the PMMA film was adjusted to be ∼18 chains/μm^3^. More detailed information about the general sample preparation can be found elsewhere.^19^ Aggregation by SVA was realized by annealing for 45 minutes with different acetone and chloroform vapor ratios. The long annealing time ensures that the equilibrium of the SVA process is reached.^12^ Acetone acts as a bad solvent and chloroform as a good one for PFO. After annealing, the PMMA film was dried under nitrogen flow, resulting in fixed and isolated aggregates, which are well separated from each other (∼0.3–0.6 aggregates/μm^3^). The aggregates were investigated by exciting them one at a time and recording the PL spectra with a confocal microscope as described elsewhere.^20^ All following measurements were performed under a nitrogen atmosphere. Knowing the starting concentration before SVA and counting the luminescence spots corresponding to the aggregates and the remaining isolated polymer chains after SVA, an estimation of the average size of the aggregates is possible.^12^, ^19^

Measuring the PL spectrum of single chains before and of single aggregates after annealing can reveal the phase transition induced by SVA. The PL spectrum is a convenient observable, since the phase transition is accompanied by a red‐shift of the electronic transition and a change in the relative intensity of the vibronic transitions. The left side of Figure 2a shows the PL spectra of 124 single chains normalized and sorted in increasing order by the ratio between the first vibronic (0‐1) and electronic (0‐0) transitions. In all PL spectra, the 0‐0 peak lies around 411 nm, which is a characteristic of the α‐phase. On the right side of the figure, the sum spectra of 5 % of the single molecules with low (black spectrum) and high (grey spectrum) 0‐1/0‐0 peak ratio are shown in comparison. Both spectra have the same 0‐0 peak position, implying that there is no correlation between peak position and peak ratio. The scatter in the peak ratios can be attributed to different degrees of bending in the polymer chains.^21^, ^22^ This effect has been demonstrated in the curved chromophores of polygon model oligomers and is explained by a decreasing transition dipole moment of the electronic 0‐0 transition upon bending.^23^ At the same time, the vibronic transitions gain oscillator strength via the Renner‐Herzberg‐Teller (RHT) effect, leading to a higher 0‐1/0‐0 peak ratio in bent polymers.^21^, ^22^

**Figure 2:**
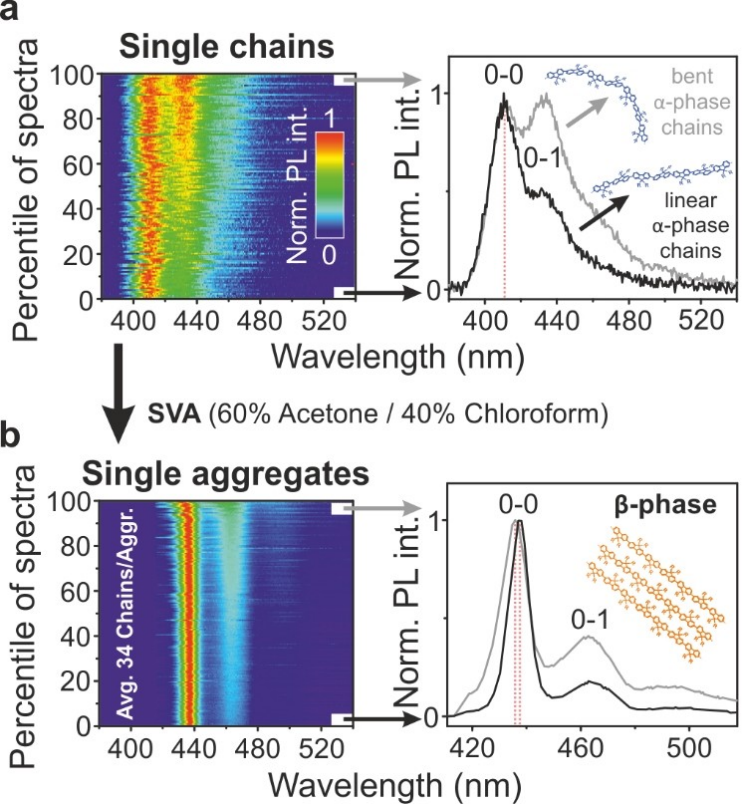
(a) On the left side normalized spectra of 124 single chains are plotted on a false‐color intensity scale. The spectra are sorted in increasing order by their 0‐1/0‐0 peak ratio. In all single chains solely the α‐phase is present. 5 % sub‐populations with low (black) and high peak ratio (grey) are summed up and shown on the right side. Different morphologies of the chain contribute to the scatter of the ratio. (b) Aggregates are formed with an average size of 34 chains per aggregate by SVA with a 60 % acetone and 40 % chloroform vapor mixture. All 173 aggregate spectra show a red‐shift compared to the single chain spectra, which implies β‐phase formation in every single aggregate. The 0‐1/0‐0 peak ratio, as well as the scatter of the ratio, is decreased in comparison to the α‐phase. A weak correlation exists between peak position and peak ratio. This correlation carries the signature of an intrachain J‐aggregate rather than an interchain H‐aggregate, which would show a suppression of the 0‐0 transition with increasing red‐shift.

During annealing with a mixture of 60 % acetone and 40 % chloroform (60/40) vapor for 45 minutes, aggregation of the PFO chains occurs. An average size of 34 chains per aggregate can be estimated by simply counting the number of luminescence spots in the microscope image of the single chains prior to annealing and the aggregates after annealing.

In the PL spectra of the 173 single aggregates, a clear change occurs upon annealing as seen in Figure 2b. All measured aggregates exhibit a red‐shift of the 0‐0 peak position of roughly 27 nm with respect to the isolated α‐phase chains. This shift to ∼438 nm reveals that the interchain ordering due to the aggregation also leads to a phase transition and the formation of the more ordered intrachain β‐phase. In addition, the 0‐1/0‐0 peak ratio decreases drastically. As for the single PFO chains, a variation of the peak ratios is present. However, in contrast to the α‐phase, we now observe a weak correlation between the peak position and the peak ratios in the β‐phase aggregates: the red‐shift of the 0‐0 transition of the β‐phase correlates with a decrease in vibronic intensity. The effect is particularly pronounced in the plot on the right of Figure 2b of the averaged spectra with low and high vibronic intensities. Since we are looking at multichain aggregates rather than single chains here, we propose that it is the different overall degrees of intrachain coupling which give rise to the distribution of 0‐1/0‐0 peak ratios, and not chain bending as in the isolated α‐phase (see Figure 2a) or β‐phase^20^ single polymer chains, where no shift of the peak position can be seen.

We note that interchain coupling, i. e. H‐type aggregation, can be ruled out in this case, as opposed to other conjugated polymer materials aggregated by SVA.^13^ H‐type aggregation leads to a red‐shift of fluorescence spectra with an additional increase of the 0‐1/0‐0 peak ratio.^13^ The transition dipole moments of the individual monomers of the β‐phase effectively add up like in a J‐aggregate.^24^ Small variations in the morphology of the aggregated chains can then lead to a distribution of effective coherence lengths, which impacts the intrachain coupling strength and therefore also the peak position and peak ratio simultaneously.^25^, ^26^, ^27^

Crucially, the PL spectra imply that a phase transition to the β‐phase has occurred within every single measured aggregate. However, it is not necessary that every PFO chain in the aggregate adopts the β‐phase in order to see a β‐phase emission spectrum. If energy transfer to the energetically lower‐lying β‐phase chains is sufficiently effective, only a small percentage of all chains need to undergo a phase transition for the PL to be dominated by the β‐phase. This observation agrees with the finding that, in the absence of aggregate formation, only a small number of isolated chains undergo the transition from α‐ to β‐phase following annealing.^20^

For the PFO chains acetone constitutes a bad solvent and chloroform a good one. During SVA, the aggregates are formed by Ostwald ripening. By varying the vapor ratios, the critical radius (R_c_) for aggregate formation is changed.^12^ Increasing the percentage of the good solvent and therefore increasing R_c_ results in larger aggregates. In addition, the interaction between the PFO polymer and the solvent molecules must be considered. Measurements of PFO fluorescence in a good/bad solvent mixture showed that if the good solvent prevails, the chains remain in the α‐phase.^28^ By increasing the proportion of the bad solvent, the chains tend to agglomerate, and the β‐phase becomes visible. The phase transition arises due to the specific packing of the side chains, which induce a coplanar arrangement of the PFO monomer units.^28^, ^29^ For this reason, we test whether the size of the aggregates and the interaction between the polymer chain and the solvent molecules plays a crucial role in the phase transition during aggregation. To do this, we changed the vapor ratio and measured the impact on the phase transition in the aggregates. Ideally, a manipulation of the phase transition should become accessible by changing the vapor ratio.

Figure 3 shows aggregate spectra after annealing with different vapor ratios of acetone and chloroform. For (i) a vapor ratio of 80/20 acetone/chloroform was used, which results in an average aggregate size of 28 chains per aggregate. In all PL spectra measured, a phase transition can be identified. The scatter plot shows that the spectral variation between the single aggregates is greater compared to the sample prepared with a 60/40 vapor ratio (ii). Apparently, the chains can adopt a broader distribution of morphologies in the smaller aggregates and because the morphology is directly connected to the intrachain coupling strength, the PL spectra show a larger variability. No major differences of the PL spectra are seen between the samples prepared with a vapor ratio of 60/40 (ii) and 40/60 (iii). Overall, the spectra for the 40/60 sample (iii) tend to have slightly higher 0‐1/0‐0 peak ratios, indicating increased exciton localization and a minor decrease in the intrachain coupling strength.^25^ Since the average 0‐1/0‐0 peak ratio – which is an observable for the intrachain coupling strength^26^, ^27^ – is smallest for sample (ii), we propose that by annealing with this solvent ratio the average intrachain coupling in the aggregates becomes most effective. Even PFO chains in the β‐phase evidently show different degrees of intrachain coupling strength and SVA can be used to optimize this coupling.

**Figure 3:**
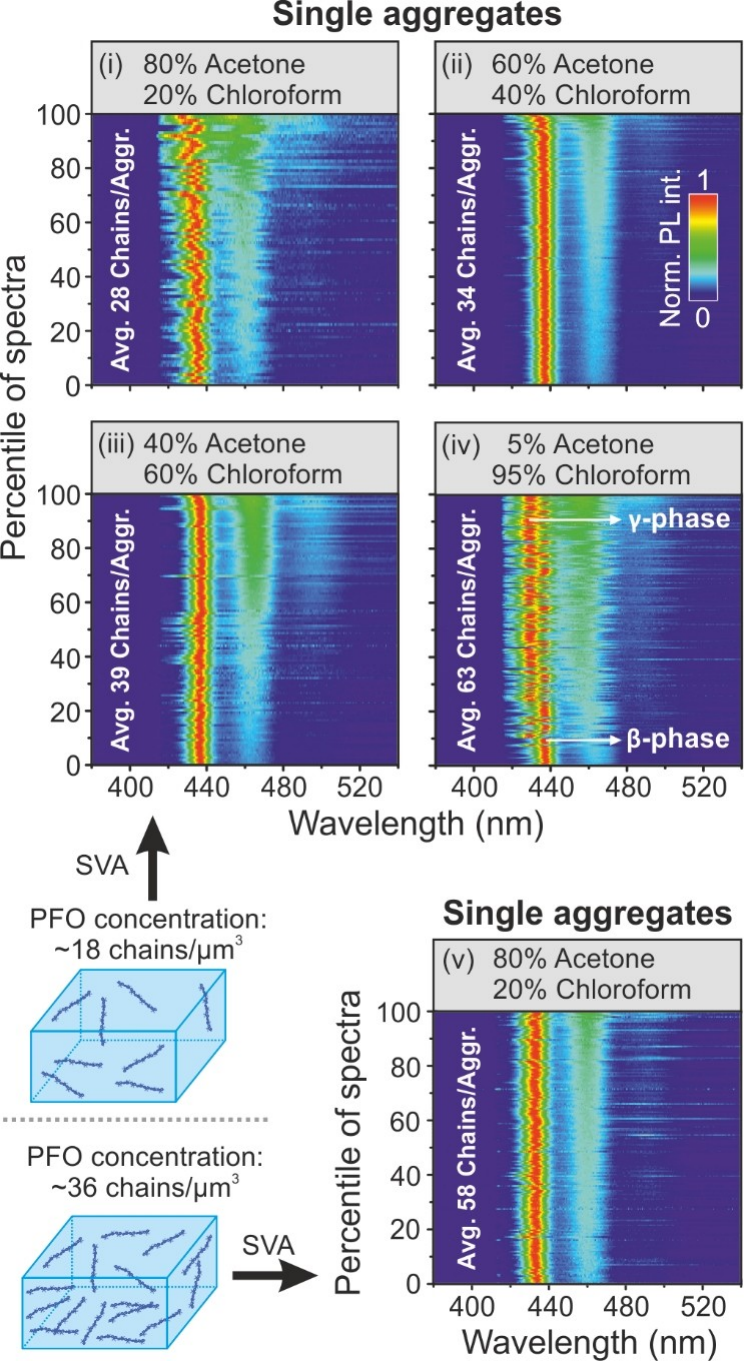
Comparison of N single aggregate spectra assembled by different acetone and chloroform vapor ratios ((i) N=87; (ii) N=173; (iii) N=110; (iv) N=210) for a starting concentration of ∼18 PFO chains/μm^3^. The average size of the aggregates increases with a higher fraction of chloroform. After SVA with 95 % chloroform, a distinct second population appears besides the β‐phase. These spectra, which show a blue shift of ∼7 nm and preferentially occur with a higher 0‐1/0‐0 peak ratio, can be assigned to the γ‐phase. By increasing the starting concentration of PFO to ∼36 chains/μm^3^ and setting the vapor ratio to 80 % acetone and 20 % chloroform we obtain aggregates ((v) N=167) that consist, on average, of ∼58 chains. This size is comparable to case (iv), but, interestingly, only β‐phase spectra arise here and no γ‐phase spectra.

As a next step, the solvent ratio was changed to 5/95 (iv) to aggregate single PFO chains. Surprisingly, under these conditions, the phase transition differs strongly in relation to the other vapor ratios: besides the β‐phase spectra with an electronic 0‐0 peak of around 438 nm, a second distinct population appears with a narrow 0‐0 peak around 430 nm. Situated between the α‐phase (411 nm) and the β‐phase (438 nm), we attribute these PL spectra to the emergence of the γ‐phase of PFO. By sorting the spectra from low to high peak ratio, it becomes apparent that the γ‐phase spectra have a larger 0‐1/0‐0 peak ratio compared to the β‐phase. This larger ratio can be attributed to the lower overall degree of intrachain coupling and thus poorer J‐type coupling within the γ‐phase. To see whether the increased aggregate size or the higher percentage of good solvent promotes the formation of the γ‐phase, we changed the starting concentration of the PFO chains to ∼36 chains/μm^3^ and performed SVA with a ratio of 80 % acetone and 20 % chloroform. The size of the aggregates formed (v) is comparable to case (iv), but interestingly no γ‐phase emission is observed. Therefore, we attribute the formation of the γ‐phase to the high ratio of chloroform. We speculate that the prevalence of the good solvent may influence the character of the aggregation. In this case, additional aggregation processes such as coalescence may also play a role besides the usual Ostwald ripening during aggregation.^30^

The two distinct populations in the PL spectra of (iv) corresponding to the different phases (green=γ‐phase; orange=β‐phase) can be clearly seen in a histogram of the 0‐0 peak positions as shown in the left panel of Figure 4. In the population, which shows only γ‐phase emission, apparently not a single chain made a transition to the β‐phase, for otherwise energy transfer to the energetically lower‐lying β‐phase chain would occur and the corresponding emission features would become visible in the spectra. The histogram also reveals that a few aggregates sit in the intermediate region between the two phases (marked red). It stands to reason that in these aggregates some PFO chains are present, which adopt the γ‐phase, whereas others adopt the β‐phase. This interpretation can be further strengthened by comparing the sum spectra of these three populations in the right panel of Figure 4.

**Figure 4:**
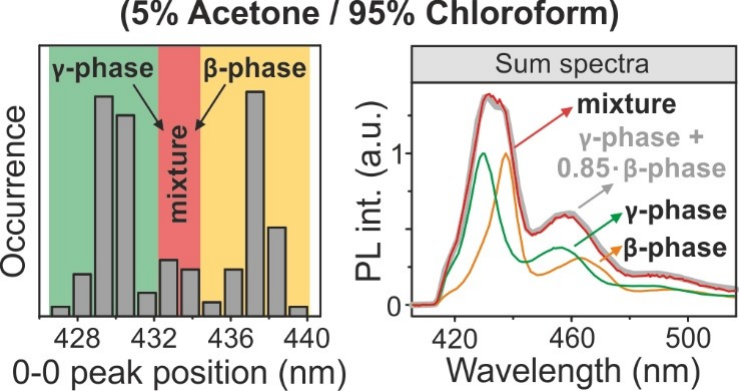
Histogram of the 0‐0 peak positions of 210 aggregates assembled with 5 % acetone and 95 % chloroform vapor ratios. Two populations corresponding to the β‐phase (orange) and the γ‐phase (green) can be distinguished. On the right a sum spectrum is shown for the aggregates marked by red in the histogram. This spectrum is described by a superposition (grey) of β‐phase (orange) and γ‐phase (green) spectra. In these aggregates both phases are clearly present.

We add up all single‐aggregate spectra corresponding to the peak positions marked by green, red and orange in the histogram and plot the sum spectra on the right side of Figure 4. The red spectrum shows features of both γ‐phase (green spectrum) and β‐phase (orange spectrum) luminescence.

Adding up these two spectra in a ratio of 100 : 85 gives the grey curve, which accurately describes the measured red spectrum. Evidently, chains of both γ‐ and β‐phase contribute to the PL emission in these aggregates, i. e. both phases coexist within one aggregate. We note that for PL from a particular phase to occur, not all chains in the aggregate need to have entered this phase: excitation energy transfer can occur from the higher‐energy α‐phase chains to the lower‐energy γ‐ or β‐phases.^31^ We conclude that the energy transfer efficiency – i. e. the interchain coupling strength – to the energetically favorable β‐phase sites must be low in these aggregates (iv) since energy transfer from the intermediate‐energy γ‐phase to the lowest‐energy β‐phase is suppressed. Such a low energy transfer efficiency could arise, if coalescence occurs during aggregation. If two aggregates showing γ‐phase and β‐phase coalesce,^30^ the coupling to the energetically lower‐lying β‐phase could be sufficiently weak so that emission from both phases becomes visible.

We have shown that a phase transition starting from the α‐phase of PFO can be induced by aggregation via SVA. For a vapor ratio of acetone/chloroform of 80/20 (i), 60/40 (ii) and 40/60 (iii) only a phase transition to the β‐phase occurs. On average, the 0–1/0‐0 peak ratio is lowest in aggregates formed with a solvent ratio of 60/40 (ii). Because the peak ratio is presumably linked to the degree of electronic delocalization, i. e. the intrachain coupling strength,^26^, ^27^ the measurements suggest that the average coupling strength has its maximum in this type of aggregates formed by an almost equal mixture of good and bad solvent. Interestingly, by applying predominantly the good solvent chloroform (5/95) as an annealing vapor, 61 % of the aggregates emerge in the γ‐phase or in a mixture of β‐ and γ‐phases. Changing the vapor ratio therefore offers a unique handle to manipulate the intrachain phase while potentially also controlling interchain ordering.^13^, ^19^ However, further exploration of the SVA induced aggregation and its impact on interchain ordering is necessary so that a correlation with the degree and duration of swelling can lead to further handles of morphological control.^32^

## Conflict of interest

The authors declare no conflict of interest.
